# Contribution of the TGFB1 Gene  to Myocardial Infarction Susceptibility 

**Published:** 2012

**Authors:** R.M. Barsova, B.V. Titov, N.A. Matveeva, A.V. Favorov, T.S. Sukhinina, R.M. Shahnovich, M.Ia. Ruda, O.O. Favorova

**Affiliations:** Pirogov Russian National Research Medical University , Moscow; Russian Cardiology Scientific and Production Center, Moscow; Vavilov Institute of General Genetics, Russian Academy of Science, Moscow; Oncology Biostatistics and Bioinformatics, Johns Hopkins School of Medicine, Baltimore, MD 21205, US

**Keywords:** myocardial infarction, Russians, genes, allelic polymorphism, transforming growth factor β1, *TGFB1*, APSampler

## Abstract

Carriage frequencies of alleles and genotypes of the*TGFB1 *gene polymorphous loci –509C>T (rs1800469), 869T>C (rs1982073), 915G>C (rs1800471), which affect the level of cytokine TGF-β1 production, were analyzed in the patients of Russian ethnic descent with myocardial infarction (MI) (406 cases) and in the control group of the same ethnic descent (198 controls). Significant association with MI was observed in carriage frequencies of the alleleTGFB1*–509T (*p*=0.046, OR =1.45, 95% CI: 1.02-2.06), genotypes TGFB1*869T/T (*p*=0.0024, OR =1.75, 95% CI: 1.22-2.51), andTGFB1*915G/G (*p*=0.048, OR=1.76, 95% CI: 1.05-2.97). Linkage disequilibrium analysis for these SNPs has shown that the associations revealed can be considered to be independent. A complex analysis of MI association with combinations of alleles/genotypes of said SNPs indicates their cumulative effect. An analysis of susceptibility to early-onset MI (≤ 50 years old) revealed a positive association of the alleleTGFB1*–509T (*p*=0.002, OR=2.24, 95% CI: 1.35-3.71) and genotypeTGFB1*869T/T (*p*=0.008, OR=1.93, 95% CI: 1.18-3.15), as well as their additivity. An analysis of susceptibility to recurrent MI revealed an association of the genotypeTGFB1*–509T/T (*p*=0.0078, OR=2.60, 95% CI: 1.28-5.28). The results obtained indicate the important role of the*TGFB1*gene in susceptibility to MI, including early-onset and recurrent MI, in Russians.

## INTRODUCTION 

The absolute majority of cardiovascular diseases (CVD) are complex polygenic diseases. To date, a lot of CVD genetic markers have been revealed; among them a special place belongs to the genes, which encode proteins involved in the atherosclerotic process. This is also true for ischemic heart disease (IHD), which includes the myocardial infarction (MI) as one of the forms. Among these markers is the *TGFB1 * gene,product of which (transforming growth factor beta 1) belongs to the TGF-β cytokine superfamily and functions both as a pro-atherogenic and anti-atherogenic factor. There are three single nucleotide polymorphisms of interest: rs1800469 (SNP –509C>T) in the promoter, rs1982073 (SNP 869T>C, Leu10Pro), and rs1800471 (SNP 915G>C, Arg25Pro) in the signal sequence (exon 1) [1]. All of the polymorphisms studied affect the level of TGF-β1 production: according to the literature [2-4], allele T of the SNP –509C>T, allele Т of the SNP 869T>C, and genotype G/G of the SNP 915G>C are associated with the higher protein plasma level. 

A number of studies are dedicated to the association analysis of these SNPs with IHD or MI development; some of them demonstrate such an association [1, 5, 6], while some do not.  

Since it was finally proved that genetic susceptibility to many polygenic diseases varies in different ethnic groups, studies should be performed in ethnically homogenous populations. No studies have been performed on the association of the SNP 869T>C and 915G>C of *TGFB1* gene with MI and other CVDs occurrence in Russians. Previously, we observed a positive association of MI occurrence in a smaller sampling with the allele * TGFB1* *–509T in combinations with the alleles/genotypes of the SNPs of other inflammation genes, as well as a contribution of the “alternative” allele –509*С to protective combinations [7]. In the present study, a search for the association of rs1800469 (SNP –509C>T), rs1982073 (SNP 869T>C, Leu10Pro), and rs1800471 (SNP 915G>C, Arg25Pro) of the *TGFB1* gene with MI occurrence was performed in more than 400 patients of Russian descent, as well as an analysis of haplotypes of these SNPs in healthy individuals. The distribution of alleles and genotypes of said SNPs were analyzed by a comparison of patients of different age groups and subjects of the control group, as well as groups of patients with a single and recurrent MI(s). 

## MATERIALS AND METHODS 

For the case-control study we used blood samples from 406 patients of Russian descent with MI, with a mean age ± standard deviation (SD) - 57.5 ± 12.8 years. 272 of the individuals were men (mean age - 53.4 ± 11.9 years), and 134 were women (65.6 ± 10.3 years). The control group consisted of 198 individuals of Russian descent without CVDs in medical history. 112 of the individuals were men (mean age - 57.1 ± 11.9 years), while 86 were women (mean age - 63.2 ± 14.2 years).  

*MI was diagnosed according to the *2001 AHA/ESC guidelines. The subjects of the control group underwent an examination in order to exclude IHD. All of the patients (or their relatives) and the subjects of the control group gave informed consent for this study. 

### The extraction of DNA

 with a phenol-chloroform mixture was performed according to [8]. 

### The polymorphous loci

 of the *TGFB1* gene were analyzed using the PCR-SSP method. A DNA fragment of 283 bp containing SNP 869T>C was amplified using the allele-specific primers 5’-AGCAGCGGTAGCAGCAGCA-3’ (SSP T), 5’-GCAGCGGTAGCAGCAGCG-3’ (SSP C), and the common primer 5’-CT ACCTTTT GCC GGGAGACC -3’. In the case of SNP 915G>C, a DNA fragment of 125 bp was amplified using the allele-specific primers 5’- TGGTGCTGACGCCTGGCCG-3’ (SSP G), 5’-TGGTGCTGACGCCTGGCCC-3’ (SSP C), and the common primer 5’-GGCGAGCCGCAGCTTGGACA-3’. All primers were designed using the Vector NT I 7.1 and Primo software [9]. The amplification mixture (10 µl) contained 70 mM Tris-HCI (pH 9.0), 20 mM (NH _4_ ) _2_ SO _4_ , 1.0 mM MgCl _2_ , 0.025% Tween-20, 0.025% NP-40, 5 pmole of each primer, 0.2 mM dNTP, 0.5 U Taq-polymerase, and 100–200 ng DNA mineral oil. Amplification program: 95°С, 5 min. Then, 10 cycles: 95°С – 1 min, 64°С – 1 min, 72°С – 1 min; and 20 cycles: 95°С – 30 s, 58°С – 50 s, 72°С – 50 s. PCR was performed in МС16 amplifier (DNA-technology, LLC, Russia). The presence of amplified products was checked by electrophoresis in a 2% agarose gel with ethidium bromide. SNP –509C>T was analyzed as described in [7]. 

**Statistical analysis**. An analysis of the deviation of the observed genotype frequencies from the Hardy–Weinberg equilibrium and the linkage disequilibrium (LD) analysis were performed using the free Haploview 4.0 software [10]. The carriage frequencies of alleles and genotypes of single SNPs in different groups were compared by a two-sided Fisher’s exact test using the free online GraphPad Instat software [11]. We used the APSampler software [12,13] to identify significant associations between MI and carriage of allele/genotype combinations of said *TGFB1* SNPs and to assess the level of association significance for each combination found by the general algorithm by the value of one-sided Fisher’s exact test. The differences were considered significant at *p-values* ≤0.05. We computed the odds ratio (OR) and its 95% confidential intervals (CI) for each allelic combination found, and we considered only those combinations, for which CI did not cross 1. 

## RESULTS 

The genomic typing of the polymorphous loci –509C>T, 869T>C and 915G>C of the *TGFB1* gene was carried out in patients with MI and individuals without CVDs, followed by an analysis of a possible association of these SNPs with MI development. We did not observe deviations in the distribution of SNPs alleles and genotypes frequencies from the Hardy–Weinberg equilibrium in the control group. In the group of patients, SNP 869T>C and 915G>C were in the Hardy–Weinberg equilibrium, while –509C>T was not ( *p* = 0.0007). 

**Fig. 1:**
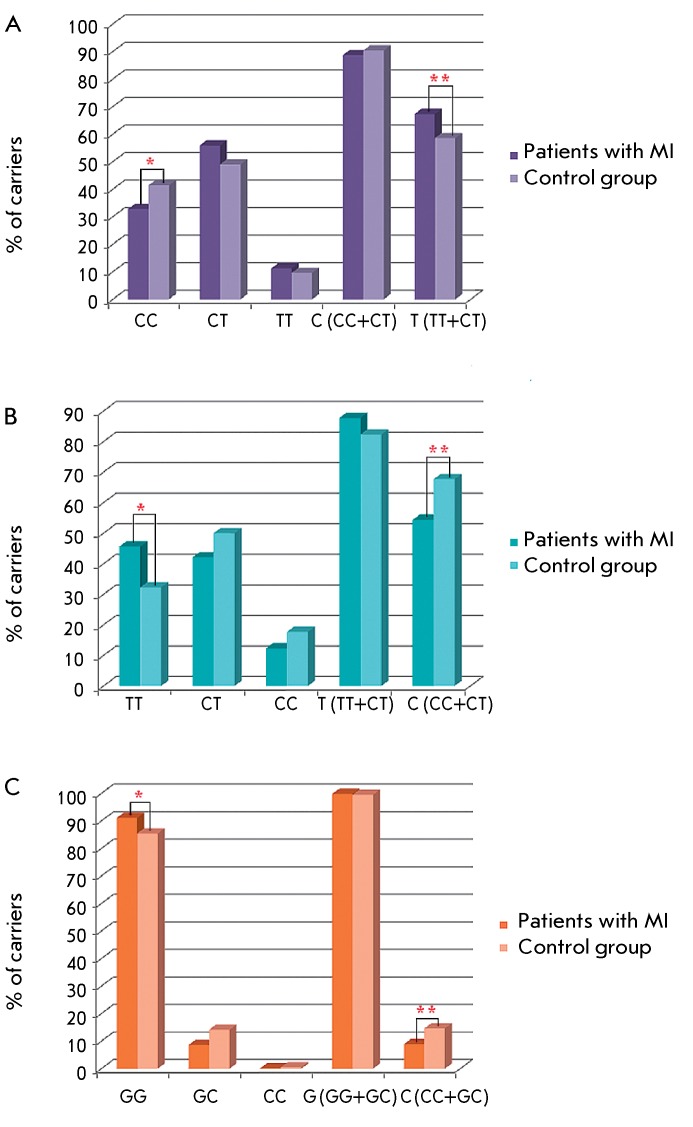
Carriage frequencies of alleles and genotypes of *TGFB1* gene polymorphous lociin the patients with MI and in the control group. A, SNP –509C>T. * *p * = 0.046, OR = 0.69; ** p = 0.046, OR = 1.45; B, SNP 869T>C. * *p * = 0.0024, OR = 1.75, ** *p * = 0.0024, OR = 0.57; C, SNP 915G>C. * *p * = 0.048, OR = 1.76; ** *p * = 0.048, OR = 0.57.

The carriage frequencies of alleles and genotypes of the *TGFB1* gene in the patients and controls are shown in *Fig. 1* . The allele *TGFB1* *–509T (as genotypes Т/Т and С/Т) was more frequent in the group of patients ( *p* = 0.046, OR = 1.45, 95% CI: 1.02–2.06) than in controls, while the genotype *TGFB1* *–509C/C was less frequent ( *p* = 0.046, OR = 0.69, 95% CI: 0.49–0.98). Furthermore, genotypes *TGFB1* *869T/T ( *p* = 0.0024, OR = 1.75, 95% CI: 1.22–2.51) and *TGFB1* *915G/G (p = 0.048, OR = 1.76, 95% CI: 1.05–2.97) were also more frequent in the group of patients. Accordingly, alleles *TGFB1* *869C (sum of С/С and С/Т genotypes) ( *p* = 0.0024, OR = 0.57, 95% CI: 0.40–0.81) and *TGFB1* *915C (sum of С/С and G/C) ( *p* = 0.048, OR = 0.57, 95% CI: 0.34–0.96) were more frequent in the control group. Thus, carriage of the allele *TGFB1* *–509T, or genotype *TGFB1* *869T/T, or genotype *TGFB1* *915G/G, can be considered as risk factors for MI development. Thereby, an association of the genotype *TGFB1* *869T/T with MI development is 20 times more significant than for other markers. 

**Table:** Association of myocardial infarction with carriage of alleles/genotypes of three *TGFB1* SNPs

SNPs of*TGFB1 *gene			Carriers (%)/non-carriers (%) of the combination		*p*-value in comparison of frequencies*	OR (95%CI) for reliable differences
–509C>T	869T>C	915G>C	Patients(N=397)	Control group(N=198)		
Predisposing combinations						
T	T/T	G	116 (29.2)/281 (70.8)	33 (16.7)/165 (83.3)	0.00048	2.06(1.34-3.18)
T	T/T	-	116 (29.2)/281 (70.8)	33 (16.7)/165 (83.3)	0.00048	2.06(1.34-3.18)
-	T/T	G	181 (45.6)/216 (54.4)	64 (32.3)/134 (67.7)	0.0012	1.75(1.23-2.51)
T	-	G	273 (67.2)/ 133 (32.8)	116 (58.6)/ 82 (41.4)	0.023	1.45(1.02-2.06)
Protective combinations						
C	C	C	17 (4.3)/380 (95.7)	23 (11.6)/175 (88.4)	0.00097	0.34(0.18-0.65)
-	C	C	20 (5.0)/377 (95.0)	23 (11.6)/175 (88.4)	0.0036	0.40(0.22-0.75)
C	-	C	31 (7.8)/375 (92.2)	29 (14.6)/169 (85.4)	0.0061	0.48(0.28-0.83)
C	C	-	190 (47.9)/207 (52.1)	117 (59.1)/81 (40.9)	0.0062	0.64(0.45-0.90)

*For each of the three SNP, the risk allele (genotype) is shown in a darker background color than the protective allele.

**Presented in decreasing order of the significance level for predisposing and protective combinations, individually.

Due to the fact that all of the SNPs above are localized in one gene, and taking into account published data for the linkage disequilibrium of the loci in this region [14-16], we analyzed the possible haplotypes of these SNPs. We used only the control group, because only in this group Hardy–Weinberg equilibrium was observed for all SNPs. Calculation of the pairwise linkage disequilibrium between SNP –509C>T, SNP 915G>C, and 869T>C, as measured by the Haploview 4.0 software [10], revealed a weak linkage (r ^2^ < 0.05 for all pairs). Probably, the association of MI with these SNPs can be considered to be independent. It provides grounds for an analysis of the association of the alleles/genotypes-combined carriage with MI development using the results of APSampler calculations ( *Table* ). 

As seen from the *Table* , combined carriage of the allele *TGFB1* *–509T and genotype *TGFB1* *869T/T, each of which is the MI risk factor ( *Fig. 1A, B* ), leads to an increase of both the significance level ( *p* = 0.00048) and OR value (2.06) relative to the carriage of each of them alone. An addition of *TGFB1* *915G, carriage of which wasn’t associated with MI alone ( *Fig. 1B* ), to this biallelic combination maintains the *p* and OR values unchanged, since all ( *TGFB1* *–509T + *TGFB1* *869T/T) combination carriers also bear the allele *TGFB1* *915G. Carriage of the other two combinations ( *TGFB1* *869T/T + *TGFB1* *915G) and ( *TGFB1* *–509T + *TGFB1* *915G) is characterized by the less significance level ( *p* = 0.0012 and 0.023, respectively) and OR values (1.75 and 1.45, respectively) than the combination ( *TGFB1* *–509T + *TGFB1* *869T/T). 

Combined carriage of the alleles *TGFB1* *–509C, *TGFB1* *869C, and *TGFB1* *915C is also highly significant, but it is negatively associated with MI ( *Table* ). A single negative association with MI was shown for the two latter alleles, but not for *TGFB1* *–509C ( *Fig. 1* ). In this case, the significance level and OR dissimilarity from 1 for the triallelic combination ( *p* = 0.00097, OR = 0.34) were higher than for all three biallelic combinations embodied ( *p* 0.0036 to 0.0062; OR 0.40 to 0.64). Furthermore, alleles/genotypes of the three SNPs included in protective combinations are alternative to the alleles included in the predisposing combination. 

It is known that genetic risk factors are often more significant for MI development at a younger age (in the case of early-onset MI). On this basis, we analyzed the alleles/genotypes distribution of polymorphous loci in patients of different age subgroups with MI. By the allocation of the a subgroup of patients who had developed the MI before 50 years of age inclusive (121 individuals), comparison of them with the total control group showed differences similar to the differences in the total sample. Thus, the allele *TGFB1* *–509T ( *p* = 0.002, OR = 2.24, 95% CI: 1.35–3.71) and genotype *TGFB1* *869T/T ( *p* = 0.008, OR = 1.93, 95% CI: 1.18–3.15) were more frequent in the group of patients younger than 50, while the genotype *TGFB1* *–509C/С ( *p* = 0.002, OR = 0.45, 95% CI: 0.27–0.74) and allele *TGFB1* *869C ( *p* = 0.008, OR = 0.52, 95% CI: 0.32–0.85) were more frequent in the control group. Furthermore, the complex analysis revealed the combination of the allele *TGFB1* *–509T and genotype *TGFB1* *869T/T ( *p* = 0.00015, OR = 2.73, 95% CI: 1.60–4.63), which is associated with an early-onset MI and analogous to that obtained in the total group.  

Then, we divided the group of patients into subgroups of patients with first MI (73 individuals) and with recurrent MI(s) (226 individuals) and compared their genotypes with each other. We found an association with only one SNP: genotype *TGFB1* *–509T/T was more frequent in the group of recurrent MI(s), then in the group of patients with only one MI ( *p* = 0.0078, OR = 2.60, 95% CI: 1.28–5.28), whereas allele *TGFB1* *–509C was protective ( *p* = 0.016, OR = 0.38, 95% CI: 0.19–0.78). Combinations found by complex analysis were less significant than single association of SNP –509C>T. 

## DISCUSSION 

**Fig. 2:**
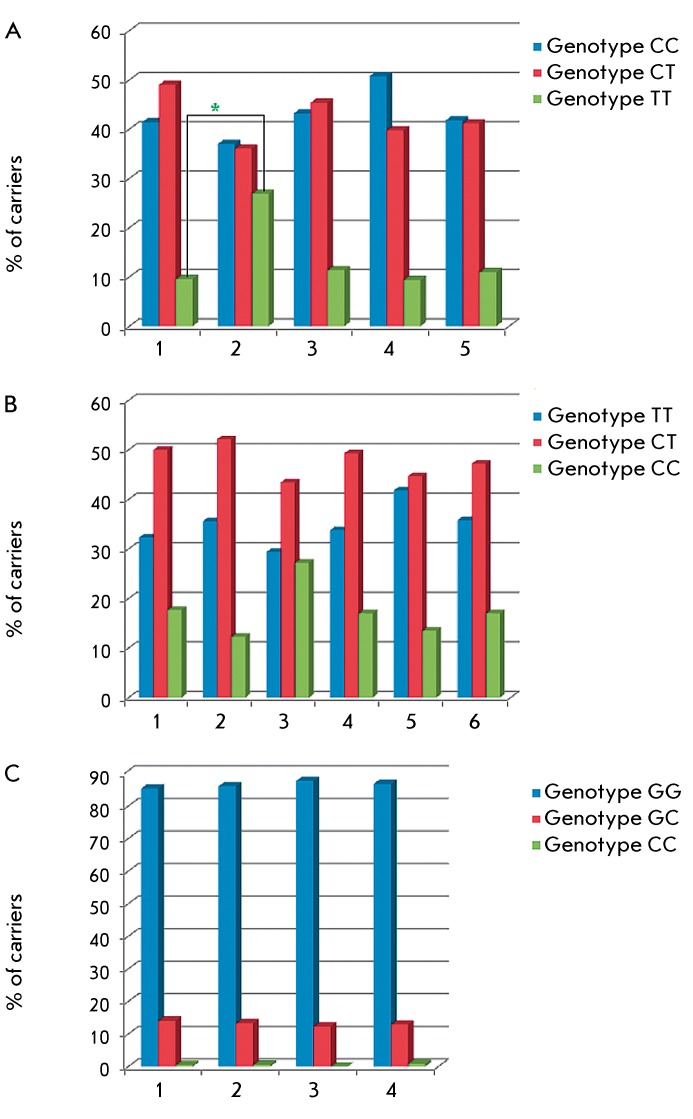
Carriage frequencies of genotypes of the *TGFB1 * gene polymorphous loci in different Caucasian populations. A, SNP –509C>T. 1 – Russia, control group in the present study, 2 – Italy [6], 3 – Germany [5], 4 – UK [14], 5 – ECTIM study (France + Northern Ireland) [1]; B, SNP 869T>C. 1– Russia, control group in the present study, 2 – Russia, men [17], 3 – Italy [6], 4 – Germany [5], 5 – England [14], 6 – ECTIM study (France + Northern Ireland) [1]; C, SNP 915G>C. 1 – Russia. Control group in the present study, 2 – Germany [5], 3 – UK [14], 4 – ECTIM study (France + Northern Ireland) [1]. * - *p* = 0.001.

In this paper we present data on the distribution of alleles and genotypes of three functional polymorphous loci of the *TGFB1* gene in a population sample of Russians (control group). We could find published data on the frequency of alleles/genotypes only for one of the SNPs studied in Russians: namely 869T>C, notably in a group of men [17]. The genotype frequencies obtained in that study are close to the frequencies identified in the present study ( *Fig. 2B* ). *Fig. 2* shows data not only for Russians, but also published data on the genotype distribution of the SNP–509C>T, SNP 869T>C and SNP 915G>C of the *TGFB1* gene in different Caucasian populations. Generally, the genotype frequencies we observed in Russians fall within a fairly wide range of frequencies, which are described for different populations of Europe. Besides, the picture varies for individual SNPs: if there is complete uniformity of genotype distribution for 915G>C in all the Caucasian populations studied ( *Fig. 2C* ), then in the case of SNP–509C>T and SNP 869T>C, ethnic differences reach the significance level ( *р* = 0.001) when the genotype frequencies of –509Т/Т in Russians and Italians are compared ( *Fig. 2A* ). 

These data may reflect differences in the linkage patterns of *TGFB1* polymorphous loci in different populations. Although many studies on Caucasians have revealed different haplotypes containing *TGFB1 * polymorphous loci, we obtained no similar data for Russians. Probably, this is due to the population-specific character of LD pattern formation, while the differences observed reflect the significant ethno-specific variability of haploblocks [18]. 

We have shown the contribution of the *TGFB1* gene, specifically the contribution of carriage of the polymorphous loci –509C>T, 869T>C and 915G>C of the *TGFB1* gene, as well as their combinations to MI genetic susceptibility in Russians. The comparison of significance level and OR values for these combinations with those of individual alleles/genotypes suggests that, in case of their combined carriage, a cumulative effect occurs, which most likely reflects summation of the independent contributions of different polymorphous loci of the same gene in MI development. Since all the risk alleles/genotypes we found ( *TGFB1* *–509T, *TGFB1* *869T/T and *TGFB1* *915G/G) are associated with the higher level of gene expression [2–4], we can assume that cumulative association is determined by the unidirectionality of changes in the TGF-β1 protein level. 

In the analysis of genetic susceptibility to early-onset MI, we observed a significant association of SNP *TGFB1* *–509C>T and 869T>C, but not for 915G>C. In the case of recurrent MIs, significant associations were observed only for SNP *TGFB1* *–509C>T. Besides, the risk alleles were the same as for the total group of patients. Thereby, SNP –509C>T and SNP 869T>C can be considered as MI markers irrespective of age, including early-onset MI, while the SNP *TGFB1* *–509C>T can serve as a predictive marker of recurrent MI development. However, we cannot exclude that the reduction in the number of associated markers in the subgroups analyzed, compared to the total group of patients, may be caused by the reduction in the size of the sample. 

The associations found are in general consistent with the results obtained for other Caucasians, although it should be noted that significant variations are found in the published data. The results of study of early-onset MI in Italians [6] and ECTIM study, which was performed on the French and the Northern Ireland population [1], are similar to our findings on the predisposing role of the alleles *TGFB1* *–509T and *TGFB1* *915G, respectively. Our results on the positive association of the allele *TGFB1* *869T coincide with the findings for Germans [5], but are in contradiction with the data for Italians [6]. A number of publications have reported no significant associations of these polymorphisms with MI in Caucasians. 

Cytokine TGF-β1, which is secreted by different cell types, including blood mononuclear cells, vascular smooth muscle cells and fibroblasts, participates in the formation and remodeling of vessels, as well as in cell differentiation and migration [19]. It plays an important role in the pathogenesis of CVDs, including atherosclerosis (including IHD and MI), essential hypertension, myocardial hypertrophy and fibrotic events, leading to heart failure and restenosis after heart surgery [20]. 

Some studies have found that TGF-β1 has an anti-atherogenic effect: it inhibits inflammation and promotes atherosclerotic plaque stabilization. On the other hand, the high level of the TGF-β1 associated with the stenosis of blood vessels and thrombosis [20] promotes fibrosis and inhibits endothelial regeneration [21]; i.e., it acts as a proatherogenic factor. In particular, it may contribute to early lipid spots by stimulating the formation of an extracellular matrix and inhibiting its degradation [20]. On the basis of data on the role of TGF-β1 in atherosclerosis pathogenesis, both low and high TGF-β1 level can be unfavorable for the development of MI, depending on the combination of other factors. One such factor may be the ethnic identity of the groups studied. 

## CONCLUSIONS 

Our data on the association of MI with alleles/genotypes of SNP *TGFB1* *–509T, *TGFB1* *869T/T and *TGFB1* *915G/G, which are associated with the higher level of gene expression [2–4], may be indicative of the dominance of the proatherogenic functions of this cytokine in MI in Russians. 

An aggregation of the results indicates the important role of the *TGFB1* gene in the formation of susceptibility to MI in Russians. It once again shows the necessity of studying the genetic factors in each ethnic group individually.  

## Abbreviations

dNTP: deoxynucleoside triphosphate
LD: linkage disequilibrium
SD: standard deviation
SNP: single nucleotide polymorphism
TGF-β1: transforming growth factor beta 1
TGFB1: gene encoding transforming growth factor beta 1
CI: confidence interval
IHD: ischemic heart disease
MI: myocardial infarction
OR: odds ratio
PCR: polymerase chain reaction
PCR-SSP: polymerase chain reaction with sequence-specific primers
CVD: cardiovascular diseases
